# Post-COVID-19 Challenges for a Sustainable Community-Based Ecotourism: A Case Study of Rural Community in Sabah, North of Borneo

**DOI:** 10.1177/21582440221138814

**Published:** 2022-12-02

**Authors:** Jakaria Dasan, Fatimah Ahmedy, Shamezah Shamsul, Elia Godoong, Coswald Stephen Sipaut, Mohammad Saffree Jeffree

**Affiliations:** 1 Faculty of Business, Economics & Accountancy, Universiti Malaysia Sabah, Kota Kinabalu, Malaysia; 2 Universiti Malaysia Sabah, Kota Kinabalu, Malaysia

**Keywords:** community-based ecotourism, business mindset, ecotourism sustainability, ecotourism post-COVID, *Tagal* Marakau Sabah

## Abstract

COVID-19 has impacted the ecotourism industry significantly. It is imperative to examine and identify the key challenges in running rural ecotourism businesses to comprehend the mindset of the working community members before re-establishing a business model during endemic phase. This study aimed to explore and identify the resources and capabilities challenges perceived by the members of community-based ecotourism located in Marakau Village, Sabah. The study, which took place in 2021, employed a qualitative method through interviews among 10 key members of the *Tagal* Marakau Association responsible for a *tagal* ecotourism business. The interview was recorded and transcribed to identify common themes of issues. There was a total of 15 emerging issues: lack of capital, lack of business know-how, shortage of hospitality skills, lack of social capital, poor marketing ability, land dispute, absence of a strategic business model, poor financial planning, poor implementation of human resource practices, inadequate infrastructures, limited customer experiences, high fish habitat maintenance, external competitors, stakeholders support, and liability as a small business venture. These issues collated into five themes of business mindset domains: knowledge, attitude, skills, aspiration, and finance (KASA-F). Identifying the perceived challenges would help to understand the mindset of working rural community members to assist in re-formulating strategic plans for business sustainability in the post-COVID era.

## Introduction

Before the COVID-19 pandemic distracted the flourishing trend of the tourism industry, the establishment of tourism-related new ventures and businesses was thriving tremendously. As the second largest state in Malaysia, located in the northern part of Borneo Island, Sabah highly invests in tourism as the third leading economic contributor leading to a high influx of tourists and indirectly boosting employment opportunities for the local people, especially those communities residing in the rural areas. The richness of Sabah state is observed from the abundance and presence of natural resources offering natural-based tourism or ecotourism. In 2019 alone, Sabah was reported to accumulate more than 8 billion in revenues from 4.2 million tourists visiting Sabah, representing an increase in overall GDP by 0.7% and the highest GDP per capita ever recorded (Domestic Tourism Survey, 2020). This highlights the importance of the tourism sector to Sabah’s economy and serves as the best site for tourists looking for ecotourism packages when visiting Malaysia.

With a population of 3.88 million (Department of Statistics Malaysia, 2020), Sabah covers a broad and diverse physiographic range and various aquatic and terrestrial habitats. Many foreign and local tourists are attracted to the topography found in Sabah, rendering Sabah a significant tourist destination in Malaysia. With the COVID-19 pandemic in 2020, the tourism industry in Sabah is among the most affected sector (Department of Statistics Malaysia, 2021). The number of international and local tourists steadily increased from 2013 to 2016. The statistics showed a decline started in the year 2020 due to COVID-19 and continued restrictions of entry to foreign travelers in the year 2021 due to emerging variant of COVID-19 at that time, leading to the arrival of 371,187 tourists only in that latter year. When the international border was opened in early 2022, various strategies were initiated to boost the tourism industry. More attention was given to reviving rural tourism since Sabah is well known for its natural sites with scenic sights. Hence, ecotourism becomes the prime focus with the full commitment and involvement of the surrounding communities.

### Community-Based Ecotourism (CBET)

Ecotourism has become one of the sources of income for the rural community leading to the creation of community-based ecotourism. Community-based ecotourism (CBET) is heavily influenced by the local community involved in the tourist administration, and many of the benefits remain within the community (Abd Rahman et al., 2014). For a CBET business, it is a fact that the business is managed and operated by the community members themselves. Hence, it is only by right that the community has to mitigate and handle the effect the business activity may bring to their surroundings. For instance, ecotourism activity has to be operated at the expense of risking environmental resources. If businesses are held without proper plan and management, it will ruin the sustainability of the business and the environment in the long run. Thus, the community is essential in ensuring that the tourism operation will not affect sustainable tourism progress.

Tourism activities in rural areas can generate and contribute substantial income to the country but must be facilitated systematically, ensuring excellent capital flow, talent, information, technology, and management factors (Xiang & Yin, 2020). By expediting the integrated development of multiple sectors in the rural areas, more employment opportunities from community-based tourism have helped stimulate national economic growth (Jaafar & Maideen, 2012). Consequently, tourism has become one of the primary financial contributors to many countries (Sharif & Lonik, 2017), including east Malaysia, where Sabah state is located.

More community participation is required for promoting tourism destinations in rural areas. In view of this, ecotourism, as a segment of tourism, integrates elements of nature-based and small-scale travel, environmental and socio-cultural impact, as well as support and participation of the local community (Mosammam et al., 2016). In other words, ecotourism promotes a healthy and equitable relationship between humans and nature as well as between the host and guest (visitors) (Phelan et al., 2020). It inspires visitors’ feeling of being cautious and caring about the importance of enjoying a natural beauty that continues to develop into a frequent visit. The enjoyment becomes meaningful if it stimulates visitors’ deep understanding of the inter-related terms related to ecotourism which are “tourism,” “sustainable development,” and ecology (Mosammam et al., 2016).

The key elements of ecological properties of ecotourism are resources, practices, and goals aiming at alleviating poverty (Kiss, 2004; W. Liu et al., 2012). The resource element is transpired through the attractiveness of nature-based ecotourism activities where the payments for ecosystem services (PES) first appeared. All behaviors are required to be sustainable and ecological for the practice element. The third element refers to the true intention of promoting ecotourism to reduce threats to the ecosystem and conserve biodiversity (Kiss, 2004). Ecotourism activities constitute dual responsibilities to the environment and for the maintenance of the cultural life of the local people (Nugroho et al., 2016; Tuohino & Hynonen, 2001), in which ecotourism added further the responsibilities as an interaction between tourists and the local population and the natural resources of the regions visited (Jamal & Stronza, 2009). Specifically, ecotourism shapes people’s attitude toward a protective environment (W. Liu et al., 2012) through the ecological experience that provides educational and informative features (Ruhanen, 2019). In other words, ecotourism acts as a sustainable development approach that goes beyond a conservation tool (Johannesen & Skonhoft, 2005), emphasizing environmental impacts, social responsibility, and investment effectiveness (Domínguez-Gómez, & González-Gómez, 2017; Martínez et al., 2019; Naidoo & Adamowicz, 2005; Yu et al., 2019).

### Sustainable Development of Ecotourism

Environmental carelessness is the major challenge in developing ecotourism attractions (Sriarkarin & Lee, 2018). Environmental carelessness is the act of human practices that pays less concern to conserving the environment. Protecting local natural and cultural diversity and promoting these features help ensure sustainable development of the ecotourism business (Osman et al., 2018). Thus, unless the community could configure the systematic planning of running the ecotourism business, will the visitors or tourists understand the uniqueness of ecotourism which is closely related to the responsibility of taking care of nature and its environment (Ocampo et al., 2018). In fact, any organization ventures in CBET should contribute to the advancement of sustainable development’s three pillars; namely, economics, environmental, and social performance (Purvis et al., 2019).

Understanding the relevant resources and their potential is crucial as prior knowledge in determining the suitability of running an ecotourism business. Value of attractions, facility management, environmental concern, ecotourism activities, and community participation are among the elements to be found in any ecotourism potential (Tseng et al., 2016). Regarding the value attractions, the uniqueness of resources is expected (Qu et al., 2011; Yan et al., 2017), such as flora and fauna, white sandy and pristine beaches, and long and winding natural riverbanks. This uniqueness forms the value-added features of the ecotourism business. Facility management includes the infrastructures to support the operation of ecotourism, such as the modern kiosk and the toilet facility (Sriarkarin & Lee, 2018; Yan et al., 2017). The establishment of these facilities must complement nature preservation efforts. The environment may also experience degradation because of frequent exposure to visitors. Hence, ecotourism development needs to consider financial allocation for preservation. Otherwise, the long-term benefits of ecotourism may decrease (Pulido-Fernández et al., 2019; Tseng et al., 2016).

Programs arranged by the community must comprise education-oriented activities that embed the idea or concept of appreciating nature (Ocampo et al., 2018). The community acting as the primary stakeholder must be responsible for sustaining the ecotourism business. It should not be solely focused on profit-driven motivation but channel its concern on adequately managing the ecological and natural resources to safeguard the business (Masud et al., 2017; Rawlins & Westby, 2013). While community participation is crucial in managing the long-term sustainability of the ecotourism business, its role in nurturing a community mindset not to harm the environment is also warranted (Masud et al., 2017; Osman et al., 2018).

Osman et al. (2018) argued that the full involvement of other stakeholders, especially the relevant government authorities, might strengthen further the effort to avoid the destruction of the environment that characterized the ecotourism business. Insufficient understanding of ecotourism and lack of industry commitment cause inefficiency in meeting the advocated agenda (Masud et al., 2017). Lacking effective collaboration among relevant stakeholders, well-integrated ecotourism plans, lack of community participation, and weak institutional arrangements lead to unsuccessful projects (Palmer & Chuamuangphan, 2018). Community participation that empowers local control plays a vital role in ecotourism development and management (Masud et al., 2017; Palmer & Chuamuangphan, 2018). Challenges originating from the management and stakeholders in the form of mistrust, misunderstanding, and lack of transparent communication could lead to depleted resources resulting in environmental destruction (Wondirad et al., 2020). Hence, understanding the drawbacks of collaboration may prepare the remedies ahead of time.

In brief, the characteristics of the environment create the attractiveness of the destination in which tourists not only perceive satisfaction with visual contacts but authentic experiences from the environment. Nevertheless, social capital plays an essential role in improving cooperation and coordination of the local community to develop community-based ecotourism (J. Liu et al., 2014). The stronger the social capital, the higher the level of community participation in sustainable use and management of natural resources. On the contrary, if the social capital is weak, community participation will be difficult to establish.

Communities that venture into ecotourism create various business opportunities through manufactured resources, including education, knowledge interpretation, experience programs, transportation, accommodation, restaurants, convenience facilities, recreational facilities, and infrastructure, which render the destinations more attractive (Choi et al., 2021; Mai & Smith, 2015). Realizing the opportunities, more rural communities in Sabah initiated innovations using their environmental and manufactured resources to supply unique and attractive products to tourists. Environmental and manufactured resources are the primary source of ecotourism to promote attractiveness (Choi et al., 2021; Mai & Smith, 2015; Nguyen & Bosch, 2013). The International Ecotourism Society (TIES) refers ecotourism to as “responsible travel to natural areas that conserve the environment, sustains the well-being of the local people and involves interpretation and education.” This definition is in line with the idea that the community members manage the business as both the entrepreneur and enterprise, requiring them to acquire relevant knowledge and skills for pursuing the common good (Peredo & Chrisman, 2006). Community-based businesses are meant to improve the overall socioeconomic development by generating higher income and work opportunities for community members to dwell (Peredo & Chrisman, 2006).

### The Regulating Theory of Resource-Based View

A business mindset has to gear toward generating a business model that works, and contextual factors must be considered. A business model must emphasize the value it intends to create but be tallied with the organization’s capability to deliver that value. Resource-Based View (RBV) theory generates how an organization’s internal resources and capabilities can create value (Hart & Dowell 2011; Lavie, 2007). However, the resources and abilities would not be advantageous if the organization did not utilize its four main attributes—valuable, rare, inimitable, and non-substitutable, termed VRIN (Newbert, 2007). Both resources and capability play significant roles in ensuring that the organization can stand the competition from others delivering the same value. The value contribution must be matched to value appropriation to enhance the resource and capability. In other words, resource contribution must garner appropriate benefit and this address the gap in the missing “value creation-appropriation correspondence.”

Any organization is said to possess a competitive advantage provided it identifies resources or capabilities as valuable, rare, inimitable, and non-substitutable. In other words, this RVB-VRIN represents a model that an organization can pursue competitive advantages. Nevertheless, the organization must ensure that the value remains strongly demanded. Thus, VRIO was proposed as valuable, rare, inimitable, and organized (Barney & Mackey, 2016). The RBV-VRIO relies on how an organization manages and organizes the systems, structures, and processes under its control while exploiting its capability for resources. The RBV-VRIO model fosters identifying an organization’s strengths, weaknesses, and potential resources and abilities to regain or maintain competitiveness. Notwithstanding, maintaining the value’s competitiveness warrants the organization to consider three possibilities. First, an organization must not confine itself to a singular resource or capability, especially in the dynamicity of market change. Second, strategic value capitalization must align with external factors. The third consideration is flexibility in applying the value created in different places or contexts. The mindset must generate a business model that works, and contextual factors must be considered.

While efforts to combat the endemic phase of COVID-19 are ongoing, Malaysia National Tourism Policy 2020–-2030 was formulated and implemented accordingly. This policy asserts that the country needs to break out of its comfort zone with a clear message that COVID-19 should not be seen as a limitation but as a wake-up call for the country to reinvent the tourism policy as well as the strategies for becoming a competitive tourism provider among ASEAN countries toward globalization. Consequently, tourism Malaysia adopted six transformation strategies called visionary pillars, one of which is to practice and implement sustainable and responsible tourism (Tourism Malaysia, 2020). Thus, Sabah has quickly adapted itself to this transformation strategy.

Exploring and evaluating competitive advantages among small businesses and their performances are still underway. Yet, a comprehensive business model helps to sustain these small businesses, especially in community-based ecotourism. CBET is viewed as innovative, focused entrepreneurs but facing various challenges due to the lack of understanding in the business resources and management and the recent pandemic issue that caused interruption of the ecotourism industry in general. The threat stemming from external market challenges may further affect the sustainability of community-based businesses. Hence, identifying the critical issues community-based businesses face may help comprehend their employees’ mindset before a comprehensive business model can be developed. These issues would help the organization formulate strategic plans to ensure the sustainability of the community-based business, including ecotourism. This research aimed to explore and identify the resources and capabilities challenges perceived by the members of community-based ecotourism located in Marakau Village, Sabah. Identifying the perceived issues would help to understand the business mindset among the rural community members. The shifting from traditional activities into commercial activities requires mind-setting and appropriate capacity building. In particular, the workforce or the human resource needs to adapt well to the business environment. In addition to a systematic way of handling the business, openness to changes that take place will enable the CBET to stay competitive.

### Background of Tagal Marakau, Sabah

The Marakau Village is located in the district of Ranau, approximately 100 km from the capital city of Sabah, Kota Kinabalu. It takes around 2.5 hours to drive from the city. The tagal in the village was established in 2000 by the community in Marakau Village with the primary objective to protect and conserve the Mansahaban River and the fish *habitat.* The word tagal comes from the local ethnicity of Dusun which means “to hold” or “to prohibit” from catching any fish in the marked river of Sungai Mansahaban. Literally, tagal means “do not,” which prohibits or bans anyone from taking any form of life from protected rivers within a specified period. The idea is to ensure the sustainability of freshwater resources and to keep the river from being polluted or threatened by any form of destruction approach for fishing purposes. Nevertheless, fish harvesting is permitted at *tagal* but only with the permission of the local community. This *tagal* has been practiced by the Kadazandusun community for hundreds of years to preserve and conserve the life form of Sabah’s River from extinction. Several rivers with *tagal* have grown in the past decade and served as tourism attractions. Communities have transformed the *tagal* into a “fish spa” and found it to be a unique experience sought by tourists. Consequently, not only does the *tagal* practice generate income for the community, but it also manages to protect the river’s ecosystem by reducing aggressive fishing that might lead to the depletion of river sources.

*Hence*, the community has officially established an association for the tagal led by a local chairman with almost 50 registered community members, called Tagal Marakau Association. The uniqueness of the tagal and its fish habitat display has rendered it a tourist spot. The fish habitat is expanded into a “fish spa” where the sucking actions by numbers of tamed fish in the tagal have created massage actions on the immersed feet and distal lower legs. Here lie the opportunities for income generation through the tagal business. However, despite having an impressive number of community members in the association, the experiences and knowledge in managing and handling ecotourism business need to be evaluated because the expected income revenue was not met. The tagal business had to request capital injection from other stakeholders to face interrupted business from the COVID-19 threat. In October 2019, a memorandum of understanding was signed between the Sabah Fisheries Department and the *Tagal* Association of the Marakau village to manage the *tagal* system within their respective areas. At the same time, the Sabah Fisheries Department would provide technical training, equipment support, infrastructure assistance, monitoring performance, marketing promotion, and experts. Such a strong partnership might not yield the expected output without active cooperation and understanding from the business community members. It is crucial to embed the right business mindset to ensure sustainability. One focus that needs to be prioritized is the human resource domain. We are seeing a reducing figure of community members in the local business as many opted for urban and city-based employment. Hence, many existing working community members are ageing, leading to a less effective workforce with limited opportunities for business expansion.

## Method

### Sampling

Since this study utilized respondents from the association that bear the responsibilities for handling the business operation of the tagal since 2019, subjects were recruited using purposive, convenience sampling rather than randomized type. The researchers engaged with the community in the early months of 2020, when COVID-19 was not considered a major threat. These engagements yielded trust from the community involved in the study. However, with the pandemic threat from COVID-19 with movement restrictions implemented for all aspects of business sectors, online approaches were conducted several times between researchers and the community members from mid-2020 until the end of 2021. The interview finally took place sometime in November 2021, and at the time of the study, there were 46 members registered under the *Tagal* Marakau Association.

However, more than halved were mainly interested in being ordinary members without active participation or roles in the *Tagal* Marakau ecotourism business. Hence, the study recruitment gathered 10 members actively involved in manning the business and agreed to participate in the interviews so that the perceptions and information delivered would accurately reflect the contextual circumstances. They were the chairman, the secretary, the bursar, and seven others. They are among the full-time employees and active volunteer members being paid by the association to take care of the maintenance of the *tagal* as well as to entertain the visitors (Table 1). Their responsibilities and tasks justified their selection as respondents.

**Table 1.** Demographics of Respondents Interviewed in the Study.

Respondent	Age (years)	Employment status	Educational status
1	44	Employed at *Tagal* Marakau	Completed primary school
2	52	Employed at *Tagal* Marakau	Completed primary school
3	38	Employed at *Tagal* Marakau	Completed secondary school
4	47	Employed at *Tagal* Marakau	Completed primary school
5	51	Employed at *Tagal* Marakau	Completed primary school
6	39	Employed at another agency and *Tagal* Marakau	Completed secondary school
7	46	Volunteer at *Tagal* Marakau	Completed primary school
8	41	Employed at another agency and *Tagal* Marakau	Completed secondary school
9	53	Volunteer at *Tagal* Marakau	Completed primary school
10	37	Employed at another agency	Completed secondary school

The interview was conducted in Malay, the primary communication medium in this region. Out of 10 members, only five were actively employed under the *tagal* business at the studied village. The others were either employed at other agencies or not employed at the time of the study. All respondents are male and married. This sampling method was adapted from a study by Kunjuraman (2020) that explored the empowerment indicators gained by the local community from the economic, socio-cultural, and environmental aspects through its active participation in CBET located in Kinabatangan, Sabah (Kunjuraman, 2020).

## Study Design, Instrument, and Data Analysis

The study employed a qualitative method through interviews in which thematic analysis was applied after data collection. The interview was conducted either through phone or video call, whichever was convenient for the respondents. The interview was recorded and transcribed for searching emerging issues. These issues were then collated into identifying common themes for subsequent mapping (Braun & Clarke, 2006). Based on the mapping, themes were displayed in an infographic manner.

Table 2 displays the open-ended questions that the study utilized for the interview session. Before the interview, researchers had arranged an online workshop involving the research group and the core members of the studied community association. From that platform, some key points emerged and became the themes for preparing the main qualitative interview questions.

**Table 2.** The Semi-Structured Interview.

Question no.	Questions
1	What challenges did the community face when it first decided to move into a commercial *tagal* type from a traditional *tagal* type?
2	What are the current issues faced by the *tagal* Marakau community to maintain the business?
3	What main changes would you like to have in running the business?

## Results

**Figure 1.**
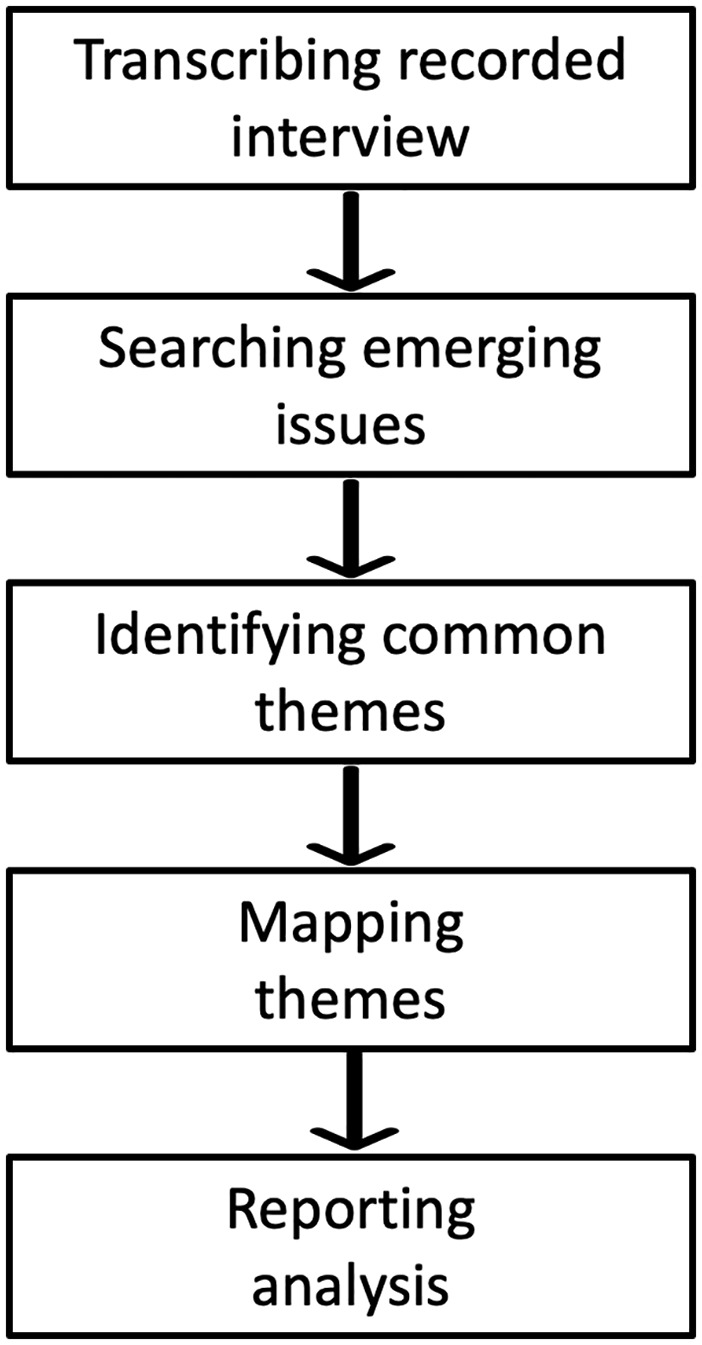
Outline of thematic analysis implemented in the study.

Figure 1 outlines the process for conducting the thematic analysis after the completion of data collection through the interview process. The result section includes the emerging issues, theme identification, and mapping report. Subsequent dissection of themes is placed under the Section “Discussion” to facilitate appraisal of previously established works. Table 3 shows some of the direct quotations received from the respondents in the interview.

**Table 3.** Excerpts of Quotations From All Respondents in the Interview.

Respondent	Interview questions		
	Challenges faced when tagal becomes commercial	Current issues faced in maintaining the business	Main changes expected to run the business
1	“Set up working hours”	“Land dispute bothers us”	“Fixed working schedule”
2	“Allowance (salary) is not fixed”	“Lack of complimenting business packages”	“Fixed amount of salary should be implemented”
3	“No sufficient fund to manage the business”	“No job roster due to a voluntary basis”	“Government should provide special funds supporting the unique ecotourism business”
4	“Lack of funds to promote its existence”	“No specific business model is used to craft the success of the business”	“Must quickly craft its expansion plan to stay relevant”
5	“Lack of infrastructure to enhance the presentation of the business”	“Even though the community has the social media account, the members do not monitor it continuously”	“Management also needs to increase their capability to run the business and be more creative”
6	“Appointment or selection of staff to man the business is not based on the relevant know-how or skills”	“Lack of funding to expand the business or widening the marketing strategy”	“t is time to apply automated tools to ease visitors’ visits to Marakau Ranau”
7	“Do not have any idea what best highlight the business to attract more visitors or tourists”	“Limited communication since staff cannot converse in English or other international languages”	“Improve further the external environment”
8	“Am not sure on the number of staff to be included in the business implementation”	“Same scripts are used every time different groups of employees come for their visit”	“Should learn Chinese, Korean, & Japanese”
9	“Staff solely depend on En Hamdan (the association’s chairman) to tell them what to do next”	“Staff are getting used to simplifying the order and not keen to voice out what bothers them”	“May consider shift or job rotation”
10	“Do not expect that the business is expanding”	“Staff feels harmonious with their job, but the feeling may not sustain due to the continuous visit”	“Perhaps a management company should be formed to run the business profitably and respectfully”

### Emerging Issues

A total of 15 themes of issues were identified from the interviews: (1) lack of capital, (2) lack of business know-how, (3) shortage of hospitality skills, (4) lack of social capital, (5) poor marketing ability, (6) land dispute, (7) absence of a strategic business model, (8) poor financial planning, (9) poor implementation of human resource practices, (10) poor infrastructures, (11) limited customer experiences, (12) high fish habitat maintenance, (13) external competitors, (14) stakeholders (government) support, and (15) liability as a small business venture.

#### Theme 1: Lack of capital

The community depends on the sole profit from the derived fund of entrance fee to the *Tagal* Marakau. The profit is not enough to pay a compatible salary to the staff, buy food for the fish, and cover other maintenance costs. When COVID-19 struck, the condition became worse. Thus, lack of capital causes the operation to be under-managed, in which shortage of funds does not coincide with the idea of expanding the business. This issue has been the greatest challenge to *Tagal* Marakau’s development.

#### Theme 2: Lack of business know-how

The community members rely only on their closeness to the tradition of managing *tagal* for a long time. Yet, when they transformed the traditional activity into a commercial-oriented business, they were unaware of the business and human resource practices required to run a community business. In other words, the know-how in managing *tagal* among the designated communities is limited. Technical knowledge is still needed to run the daily business operations profitably and adequately. Without proper planning and management, the limited funding and the available resources will quickly deplete.

#### Theme 3: Shortage of hospitality skills

The ecotourism business requires employees to have good public relations skills. Among others, tourist operators and the frontline staff must understand how to converse in English as an international language. Community-based ecotourism should seriously look at barriers like languages to attract foreign tourists. Through this language competency, operators and employees can show their hospitality to tourists.

#### Theme 4: Lack of social capital

The employees’ psychology, engagement, and working circumstances must be addressed because this affect employee performance impacting the overall business performance. Noteworthy, driven employees are more likely to fare better at the workplace. When employees are acknowledged and recognized, they feel valued. Recognition, commonly viewed as higher than a salary rise, is one of the most effective methods to inspire working members.

#### Theme 5: Poor marketing ability

Even though *Tagal* Marakau has been included in the general tourism map of Sabah, the place still does not have a proper promotion. The number of tourists visiting the site is relatively small, and no revenue was generated. No sound initiative was crafted and implemented to attract regular visitors. *Tagal* Marakau does not have the team or the designated person in the marketing. Besides relying on tourist guides or visitors who came across social media and word of mouth, no systematic planning is written for action. Not only do they need to work out the marketing strategy, but they also need to adapt well to the latest marketing tool applying technology, particularly the use of apps, and widen social media coverage.

#### Theme 6: Land dispute

The land issue is among the institutional problems in a community. *Tagal* Marakau also faces such an issue. Nevertheless, the disputed area only involved a small part of the land near the entrance of the *Tagal* Marakau. There were claims made by the next of kin or other parties when the development encroached on the “vague” area of the land. The land dispute has been reported to relevant agencies, but no final decision has been made. The case is still pending, and there is no prohibition for the business to continue in operation. Nevertheless, the association perceived such pending decisions as distracting their motivation to run the community-based ecotourism business. The slow coordination of the relevant government agencies is annoying.

#### Theme 7: Absence of strategic business model

Without a comprehensive business plan, the direction for achievable strategic objectives will not be precise. It should include a business overview, operations plan, market analysis, products and services offer, sales and marketing planning, competitive analysis, management team, financial plan, and projection. The marketing approach, for instance, should pay particular attention to the international perspectives since most tourists are unfamiliar with the uniqueness of the products offered. *Tagal* is still considered a new and relatively unknown product. Its legitimacy is yet to be established before intriguing people to visit and pay. Concerning this, the business identity of Marakau Village as a *tagal* operator needs to be recognized.

#### Theme 8: Poor financial planning

A weak understanding of the role of financial planning has caused many failures for small enterprises to achieve growth and expansion. Being inadequate to the external economic conditions, not considering the development of facilities and other means for increasing profit and sticking to the same method in developing various plans have caused failures in many enterprises, especially the small ones. Considering these factors, the association must strategize their business development accordingly. First, it has to increase the business’s income sources to support the maintenance of the fish spa. Second, the facility requires improvement that entices people to stay longer and linger in the area. Third, the community needs to work on a different plan when there are challenges from external forces.

#### Theme 9: Poor implementation of human resource practices

When the business started, the recruitment was done through word-of-mouth without any interviews. The lack of proper process in recruitment could negatively affect both the individual job search and *Tagal* Marakau’s hiring outcome. The association does not conduct a background check, and hiring is not based on educational qualifications and work experience. The person employed has no transparent job scope and job description. The staff are hired on an irregular basis and are paid based on the attendance of visitors. When there is a large influx of visitors, more teams are called on an ad-hoc basis. Thus, no routine work schedule is implemented, and work hours are not flexible.

There were incidents when *Tagal* Marakau were understaffed during peak time due to poor communication as well many of the staff were volunteers. There is no training or career development provided, including the active members of the community. Most of the learning process is based on observing the existing practices. The compensation and benefits of the employee were either not clearly stated or not fully practiced. No benefits are offered since the business is community-oriented. Such weak compensation and benefits practice does not motivate the employees who see the job as less rewarding, because they work in shifts and on a volunteer basis in which they are needed based on unprecedented time of visit. Employees must come early and sometimes work extra hours to accommodate customers’ influx. In other words, it is not practical to schedule working hours based on a fixed schedule. Employee appraisals are not part of the practice.

The management could not evaluate the employee’s performance, whereby the employee would not be motivated to work diligently, thus, self-efficacy is lacking. Furthermore, the management does not know the employee’s productivity without measuring performance. Other than that, no standards or rules are assigned related to working attendance and hours. According to the chairman, who also acts as the business manager, the staff come and go as they wish.

#### Theme 10: Poor infrastructures

Lack of signage, inadequate infrastructure and facilities were among the most prominent issues to *Tagal* Marakau. There was no excitement and comfort from reception to the river because the area only consisted of basic structures. When customers are in the *Tagal* area, they have no safety guidelines to follow. The public toilet was only at its bare minimum without proper maintenance and hygiene. The two huts meant for the tourists’ resting place were utilized for multiple purposes, including a registration place. There are no introductory posters or attractive signage seen at the location. The rubbish site was close to the reception and attracted flies and insects. In addition, the place is not disabled-friendly and has no special access for people with disabilities. Moreover, no handles were made at the side of the staircase for older people.

#### Theme 11: Limited customer experiences

The weather can be sweltering in the afternoon as the river is not roofed. Furthermore, the space for the fish spa was limited and against COVID-19 standard operation procedure, which requires social distancing. Therefore, when there are many visitors, they will not have ample time to experience the uniqueness of playing with the fish in the spa. Less than 20 people can occupy the area at one time. The fish spa experience was memorable. However, a long waiting time might cause customer dissatisfaction due to the small space of the site with improper waiting areas.

#### Theme 12: High maintenance overhead costing

When the pandemic hits, they no longer earn income. Considering that the fishes in the *Tagal* are the main attraction product, it is harder to maintain the fish spa and pay for other expenses such as buying fish food. Before the pandemic, the members and some villagers contributed to financing the fish food. However, the heavy impact of COVID-19 has made life difficult for the community to continue their contribution. The cost is not that cheap, and the feeding required exotic types of fish food. The fish are susceptible to their environment and require frequent observation, which is hard to do without adequate knowledge and staffing. The river water needs maintenance at the right pH level for the fish’s growth and survival; hence, the need for a regular fish inspection to ensure that bacteria do not cause infection. The fishes are at risk of being infected during breeding and fighting for survival or an act of aggression from other life forms.

#### Theme 13: External competitors

There are two types of competitors that *Tagal* Marakau has to face. First the existing *tagal* operators. The popular one in Ranau is in Luanti village, Ranau. However, this *tagal*-based ecotourism is located far from the district, which gives *Tagal* Marakau an advantage. The types of fishes and their interaction with humans are also different. Second, the accompanying attractions. *Tagal* Marakau is coming up with a diversified product to be marketed at *Tagal* Marakau. Each has its competitive advantage that will keep them from competing with one another.

#### Theme 14: Stakeholders (government) support

It is found that the Malaysia Budget Financial Report did not list the *tagal* business indicating that *Tagal* Marakau is yet to be established as an ecotourism product that meets the standard of receiving specific funds.

#### Theme 15: Liability as small business venture

When the *Tagal* Marakau started in 2019, many visitors from international and domestic areas visited the place. The place received groups of international tourists several times. That was when the association initiated permanent staff recruitment, albeit slowly, despite the liabilities of lacking the resources required to seek out new opportunities. The association was not proactive in gathering information on the challenges of community-based ecotourism in operating *tagal* businesses. As a small venture, *Tagal* Marakau may engage with the existing *tagal* operators to improve their business offerings and link themselves to established large ecotourism operators.

### Identification of Themes

**Figure 2.**
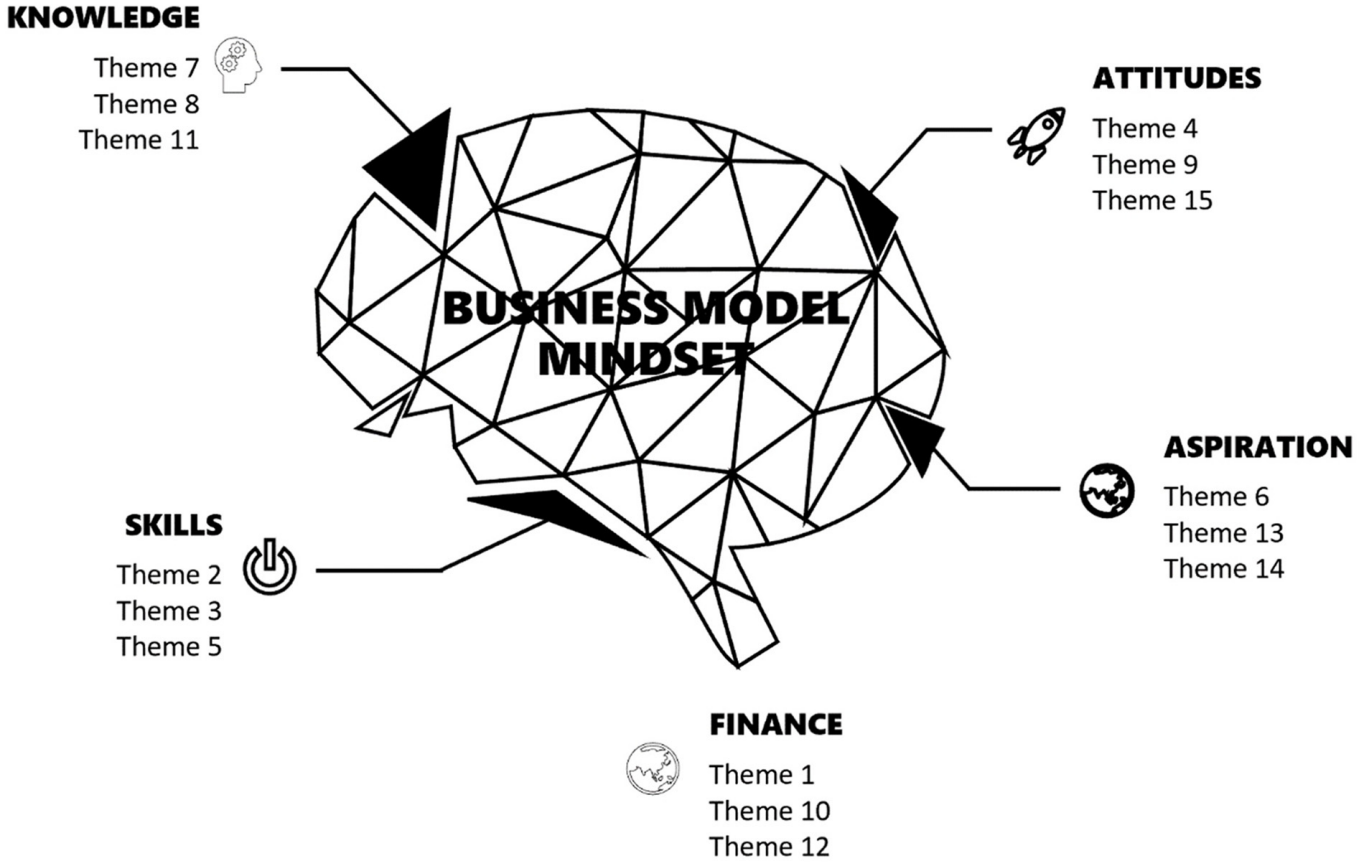
The mindset of a business model incorporates knowledge, attitude, skills, aspiration, and finance domains.

Based on the input given by the respondents, challenges were identified that mostly referred to the foreseen shortcoming of capabilities of the working members in handling ecotourism. Meanwhile, the resource issue was also quite worrying since the fish’s health may determine the business’s longevity. RVB stated that a particular company would become competitive if the resource and capability were not jeopardized. Thus, to avoid such incidents from happening, the community members’ mindset comprising of knowledge (K), attitudes (A), skills (S), and aspiration (A) have to be specifically entertained. Knowing that the *Tagal* Marakau was recently ventured into the ecotourism business, challenges related to finances must receive specific attention. These 15 emerging issues were mapped into five themes of business mindset domains: knowledge, attitude, skills, aspiration, and finance (Figure 2). Table 4 depicts the mapping of emerging issues into identified parts.

**Table 4.** Mapping of Emerging Issues into Domains of KASA-F.

Knowledge (familiarity with understanding an issue)	
Theme 7	Absence of strategic business model
Theme 8	Poor financial planning
Theme 11	Limited customer experiences.
Attitude (mind orientation)	
Theme 4	Lack of social capital
Theme 9	Poor implementation of human resource practices
Theme 15	Liability as small business venture
Skills (talent required to perform a task)	
Theme 2	Lack of business know-how
Theme 3	Shortage of hospitality skills
Theme 5	Poor marketing ability
Aspiration (motivation to achieve goals)	
Theme 6	Land dispute
Theme 13	External competitors
Theme 14	Stakeholders (government) support
Financial	
Theme 1	Lack of capital
Theme 10	Poor infrastructures
Theme 12	High maintenance overhead costing

## Discussion

The theoretical framing was based on the Resource-Based View (RVB) theory (Barney & Mackey, 2016). This theory proposed that for a business, in this case, ecotourism, to be attracted, it needs to have resources or capabilities that catch tourists’ attention. The main product of Marakau’s ecotourism business is related to this *tagal* business. In other words, the river ecosystem where *tagal* is located is the main highlight of ecotourism in Marakau, Ranau. In 2019, the community uplifted the traditional activity into a commercial business by promoting tagal to tourists. The *Tagal* Marakau Association has been running the ecotourism business since then despite encountering some challenges.

Post-COVID-19 should set a different way on how the community perceives the running of their business (CBET) which is no longer the same as before. Ting et al. (2020) and Goodwin (2016) acknowledged the community’s full involvement in community project since they have the full authority in making sure their CBET site look ideal to tourists. In order to attract tourists, the goal of the business must rely more on managing the natural resources, in this case, the sustainability of the ecology and the environment should be set as a priority while responsibly using it as income generation for the community. The community is not merely a beneficiary of tourism but instead, has a bigger role to safeguard the resources that it has. In matching this expectation, positive destination management was discussed by scholars (Goodwin, 2016: Paskova & Zelenka, 2018; Ting et al., 2020) referring to the importance of human resources in managing a sustainable community. Another thing that the community needs to be aware of is the regulation of respective behavior toward other people with the hope that caring for the ecology and the environment will provide benefits to the people, the planet, or even the establishing collaborators (Farmaki et al., 2014). In fact, giving their best to uphold the responsibility will develop a high sense of belonging to their community (Joshi et al., 2021). As a result, the strong and continuous motivation in managing Tagal Marakau Ranau as a sustainable CBET is strengthened.

Those who work in the tourism industry will have a hard time getting back into the business in post-COVID-19. This is due to the loss of visitors during the pandemic and the fact that many products, especially those involving natural resources, are not fully in place. It will be difficult for the community to be ready within a short period of time even though the border has been opened to tourists in the first quarter of the year. Furthermore, the CBET operators such as Tagal Marakau Ranau may be caught off guard on managing the uniqueness and attractiveness of their CBET since there is not enough time to plan in light of the new norm. Apart from this, the river is vulnerable to flooding like what happened before in which many of the fish were swayed away due to the strong current of the river. To avoid this from keeping on happening, some infrastructural developments are necessary for specified areas that require additional costs.

Other challenges were also expected. For instance, permitting visitors to freely enter the river to feed and interact with the tamed fish may jeopardize the fish’s safety and the ecosystem. This practice may degrade the quality of the water for bacterial and fungal infections from humans’ feet. In addition, these fishes are a bit sensitive to their surroundings too which may affect their life. Some of the fishes such as large Koi require a great deal of care and nutrition. Recent experience with COVID-19 shows that when the business is closed for a long period of time, a new business really had a hard time bearing the cost of feeding the fish. Members’ own incomes were the only source of buying the food. Hence, providing adequate food for the fish and maintaining their protected ecosystem posit a big challenge to Tagal Marakau Ranau.

Given all the said challenges, careful strategic planning has to be planned by properly managing the human resources as well as the natural resources. Strategic planning warrants the community to not only focus on the profits they may acquire but also to responsibly safeguard the resources (Goodwin, 2016; Paskova & Zelenka, 2018). On that note, strategic planning must consider the capacity building of the human resources that include both capability and capacity in managing the human resources and the natural resources. In other words, the capability and capacity of human resources need to focus on improving the knowledge, attitudes, skills, and aspirations so that the effect of its competitive advantage on human resource quality and sustainable CBET is impactful (Aquino et al., 2018; Paimin et al., 2014).

When COVID-19 hit, the challenges become more significant. Even though the study identifies the challenges faced, the proposed solutions help support the effort to conserve and preserve the business and its ecosystem. The community is aware that the *tagal* business in the studied place is relatively established. Still, the community members did not firmly emphasize the unique, non-substitutable elements of the *tagal* concept. Based on the preliminary investigation, the business might face sustainability issues due to the community’s unreadiness in understanding the ecotourism business mindset. In this case, the following paragraphs thoroughly describe the human resource capabilities of acquiring proper knowledge, strategic financial planning, adequate operational skills, motivation for aspiration, and the right attitude toward a workable business model.

### Knowledge Domain

COVID-19 has prevented global tourists from visiting the country, and the lockdown has kept the movement in strict control affecting many local businesses. A business plan is a road map to success, and the association needs to have a contingent business plan. It must not rely on a belief that *tagal* business is solely for ecotourism and heavily dependent on the fish spa. It is challenging for the association to elevate its business unless it has a specific goal. The association could ask for many parties to contribute to a business plan. Prospective investors will look for helpful information about the company’s strategy and direction in the business plan in which the interested parties may opt for direct or indirect contributions. In this context, immediate assistance refers to the act of sharing through partnership. Meanwhile, indirect contribution may be in the form of corporate social responsibility or donation to receive tax exemption by contributing to the noble effort found in the business. At the same juncture, to maintain competitiveness, *Tagal* Marakau needs to highlight their uniqueness which becomes their identity (Cardon & Stevens, 2004).

The community-based ecotourism business may face sustainable issues if the financial planning is not well prepared. Good financial planning will systematically put an organization in a good position despite the instability of the environment and the uncertainty of economic conditions (Azarenkova et al., 2017). Hence, the financial planning part should not only dwell on the maintenance of the facility, but thorough consideration of the salary and expenditure for running the business administration is imperative. Every financial goal should be aligned with the strategy to garner profit. The business operator of *tagal* in Marakau Village must work out strategies for revenue generation, cost projection, recovery, and emergency plans during and after the COVID-19 pandemic.

As part of the hospitality business, *Tagal* Marakau must adhere to the notion that fulfilling customers’ needs, wishes, and desires will make them repeat business (Sharma & Rather, 2015). A fascinating customer experience will become the unique value that visitors do not see in competitors (Berry et al., 2002). Consequently, customers stay longer, garnering more services and sales toward higher profit returns (Ihtiyar & Ahmad, 2012; Sim et al., 2006). The experience is the gap between expectation and experiences that leads to satisfaction or dissatisfaction.

### Attitude Domain

Businesses must work hard to improve working conditions, perks, and chances for employees to grow and reduce the social capital gap (Shahzadi et al., 2014). In the case of *Tagal* Marakau, the community must highlight the importance of having motivated workers since it will allow them to perform better at work and, in the long run, will help them to grow and achieve high performance. This concept contradicts the psychological contract regarding a win–win relationship between employer and employee (Cardon & Stevens, 2004). A psychological contract is an unwritten contract agreed upon by an employer and an employee related to career planning, development, and career management needs (Inkson & King, 2011). Employees place a high value on pride, personal fulfilment, and peer acknowledgment.

The psychological contract is an exchange between individuals to dedicate their work to meet the organization’s needs and help individuals develop better. Hence, *Tagal* Marakau Association must analyze each employee and utilize the psychological contract approach to boost employee performance to motivate employees.

Appropriate recruiting methods often target competent people who can contribute to the organization or, in this case, the *Tagal* Marakau business. In doing so, a thorough recruiting process is required. The five stages of the recruitment process: namely, determining recruitment needs, detailed job descriptions, finding talent through posting on social networks or job boards, conducting interviews, and finally, evaluating and offering positions. If this process is followed accordingly, the association can improve the image of the business, greater profits, cost-effectiveness, and lower staff turnover. To further enhance the employee’s skills, comprehensive training programs can be made available through online methods that employees could participate in to improve their performance in technical skills training, soft skills training, management training, safety training, and other relevant training.

Another important thing that always becomes a prime concern to employees is compensation. To encounter this challenge, the employer or the association has to develop an attractive compensation package. Engagement is a strategy for keeping volunteers committed to the organization’s objectives. Focusing on involvement and developing appropriate volunteer engagement strategies can assist the business in encouraging donators to continue contributing without demanding huge returns.

It is time for the community to manage their small ecotourism businesses as not simply a place to garner extra income but for living income. Most association members participate as part-timers, except very few assist the chairman in taking care of the business. The human factor is vital in ensuring the customers gain valuable experiences during their visit. Nonetheless, studies have found that being small and new liabilities lead to small businesses’ slow growth or failure. The employees’ competencies and commitment are crucial to the success of small businesses (Cardon & Stevens, 2004). However, the liabilities have limited the emphasis on hiring, training, development, compensation, and relations. Undermining this reality will lead to a short-term establishment of small businesses.

### Skills Domain

Cardon and Stevens (2004) demonstrated that 87% of tourists returned to the attraction site because of the hospitality they had experienced (Cardon & Stevens, 2004). Hospitality service presents the idea that service providers or employees receive valuable recognition from satisfied customers and usually promote the place to their acquaintances (Crick & Spencer, 2011; Langhorn, 2004). Given this, good interpersonal communication skills need to be embedded as necessary for employees in the labor-intensive service industry. Therefore, community-based ecotourism should recruit or train its employees to possess the skills (Chiang et al., 2008). Moreover, an employee’s notable performance will become the organizational competitive advantage.

Getting a guide from qualified trainers or training institutions is necessary to enhance the skills that form the ecotourism field’s core competency, particularly in customer relations. The members should receive training in communication or language abilities and customer service skills. They should also be given marketing training to promote ecotourism, leading to more effective management of the ecotourism business (Strasdas et al., 2007). The government has mooted the upskilling and reskilling in the ecotourism industry for post-COVID-19 revival. This strategy would produce competitive ecotourism employees. Upskilling is necessary to ensure the knowledge and skills are contemporary in which employees learn additional skills or enhance existing abilities. Meanwhile, reskilling enables the employees to acquire skills or training for a new role.

Product creation is essential in any business. The ability to outclass competitors’ products will enable the business to remain popular. *Tagal* Marakau should be able to highlight the uniqueness of the *tagal* and create related manufactured resources to make *Tagal* Marakau more attractive. In addition, the community could innovate the natural resources found in Marakau into handicrafts from natural resources like bamboo-based handicrafts. Technology-based promotion aides should be applied as these are the current approaches many organizations use to promote their products and services. Nowadays, tourists are getting used to finding information from apps and websites, for instance, social media like Instagram, Facebook, and YouTube (Gulbahar & Yildirim, 2015). The frequency of posting and promotion is necessary to get a larger demographic of potential customers to visit the location. Social media platforms include a YouTube channel, Facebook, and Instagram. The association can recruit young people from the village to instigate online marketing that will help to speed up the promotion. Promotions must also be made at a specified time and not throughout the offer. The marketing section must also be alert and attentive about the crucial days when developing a promotion. Pricing must go along with the quality and the image of the product.

### Aspiration Domain

Coordinating the relevant government departments on land issues could avoid disrupting efficient management of the *tagal*. Hence, this will not disrupt the association’s motivation to run the business effectively. The respective departments should drive efforts to intervene and reconcile any dispute in the majority’s best interest. In this case, the association’s representative for the whole community must consider the benefits of all community members in the Marakau village and the surrounding area. Ecotourism is an industry that becomes a gateway for more income generation for a community and nearby community. Instead of waiting for a pending one-sided decision, it is better to have open communication leading to a fruitful collaboration resulting in the highest cooperation among the land claimants in dispute (Karkin & Janssen, 2014).

Sustaining the business depends on how creative and innovative the community management diversifies their main product. For instance, the story-telling related to the tradition of *tagal* can make visitors worth paying the visit. Selling other excellent local-made products also enables the business to gain continuous visits. In other words, *Tagal* Marakau should not only rely on *Tagal* as the only attraction that pulls visitors to their place. Proactive contact with relevant government agencies, for example, Rural Transformation Center (RTC) and Sabah Tourism Board (STB) may keep *Tagal* Marakau aware of the current development of socio-economics programs and the latest ecotourism strategies and promotion. RTC aims to expedite rural transformation by boosting rural community socioeconomic development production, growth, and competitiveness.

Meanwhile, STB, an agency under the Ministry of Tourism, Culture and Environment of Sabah, plays its role in stimulating the ecotourism industry by suggesting to the government various mechanisms to enhance the visibility of as well as community-based ecotourism. The community must accept the reality that the government can only provide essential support. It is the community that needs to be creative and innovative in making sure their business is sustainable. Meanwhile, Sabah’s Department of Fisheries (DOF) is propelling the effort to highlight *Tagal* as one of the potential ecotourism products that can lift the community’s standard of living in the rural area.

### Finance Domain

The association must proactively seek assistance from relevant government departments or non-governmental organizations (NGOs), or they can initiate their capital injection for the time being just to ensure the stability of the new business. NGOs are constantly involved in environmental protection and are sometimes known as environmental non-governmental organizations (ENGOs). The purpose of their establishment is to assist government intervention toward improving community development (Abas & Abd Halim, 2019). For instance, the Ecotourism & Conservation Society of Malaysia (ECOMY) could be one of the non-governmental organizations dedicated to sustainable ecotourism that *Tagal* Marakau should collaborate on. This organization may provide helpful assistance to ecotourism at the initial stage and provide information on various funding opportunities. Thus, networking with them might ease the burden of identifying sources of capital (Franco & Haase, 2010).

The association can also consider applying for grants, typically for infrastructural projects or training locals to venture into a socioeconomic project successfully. The Habitat Foundation Sustainable Tourism Grant, for example, is a form of a grant that is made available for the ecotourism type of business. Another way is to work on a funding strategy. The community can contact companies or establishments and ask for sponsorship for a cause, for example, a corporate social responsibility project. Perhaps, a donation from the rest of the community could be the last resort. Musa et al. highlighted those members of the society operating *tagal* in Kiulu were willing to pay RM5 even though their wages fell below the poverty line merely to ensure their *tagal* operation would sustain (Musa et al., 2020). People are motivated to contribute if they have a sense of attachment to something valuable, such as Tagal Marakau, which has sentimental value to the community (Degasperi & Mainardes, 2017).

Coordinating the relevant government departments on land issues could avoid disrupting the efficient management of the *tagal* business. Some crucial improvements are required to make *Tagal* Marakau self-promoted. Hygiene public toilets, enough water taps for washing hands near the fish spa, and the appropriate site office. The management of every business must have a working place for the employees to work comfortably and efficiently. Not only is the *Tagal* Marakau administration office temporarily situated at the secretary’s home, but the management of the visitors is also done under the tent near the entrance. The presence of a suitable workspace and location is influential in maintaining the professionalism of employees. People should not underestimate the impact proper office facilities may have on a company or organization.

Furthermore, having good workplace facilities may help employees feel more at work, leading to improved employee performance. Given ecotourism’s importance in the economy and the potential advantages, there is a need to guarantee that the tourist sector is ecologically and economically sustainable. Collaboration with government entities such as the Department of Fisheries can help to ensure *Tagal* system management and pollution avoidance. The MoU signed between the community, and the Department is the strategic step toward making *Tagal* Marakau a sustainable ecotourism product.

### Study Limitations

As one of the first studies evaluating post-COVID-19 challenges in the CBET setting of the Sabah context, this study had several limitations. First, the sample population was small, with 10 respondents participating in the interview. However, the recruited interviewees are active working members of the studied community, and the input delivered is less likely to produce different thematic analysis outcomes if the study was to be conducted with larger sample size. Second, the interview questions are generally broad, contributing to the relatively high number of emerging issues received. Nevertheless, this can be viewed in a positive perspective where dwelling conflicts occurring in the dynamic system of the active working community can be captured. The third concern is whether COVID-19 has significantly impacted the *Tagal* Marakau business leading to the temporary reduction in the income generated from this CBET, or whether there was an existing presence of unreadiness in the business mindset of this community before the pandemic itself. Thus, this study can be improved by addressing these limitations. It is possible to capture a larger number of respondents from the community with a more objective questionnaire as a study instrument based on the KASA-F domains above. In the questionnaire, further focus can be highlighted on their existing mindset, knowledge, and attitude toward the *Tagal* Marakau with further analysis of their participation levels in the business.

## Conclusions

With the COVID-19 pandemic preventing the expansion of community-based ecotourism businesses, it is crucial to perceive the post-pandemic challenges faced and the business mindset of the operating employees. Utilizing a KASA-F model helps the management of an organization to identify and group the main challenges for prioritizing solutions to ensure the sustainability of the business. In short, each challenge identified may improve the capability of the community members to run the ecotourism efficiently and effectively. In any business, the financial aspect is a solid actor that any venture must be stable with to maintain its competitiveness and face unforeseen challenges such as pandemic COVID-19. Further works are warranted to determine which challenges and issues to target in utilizing limited available resources appropriately.
